# Emerging Pathological Engagement of Ferroptosis in Gut Diseases

**DOI:** 10.1155/2021/4246255

**Published:** 2021-10-25

**Authors:** Weihua Gao, Ting Zhang, Hao Wu

**Affiliations:** ^1^Hubei Hongshan Laboratory, Wuhan, Hubei 430070, China; ^2^State Key Laboratory of Agricultural Microbiology, College of Animal Science and Technology, Huazhong Agricultural University, Wuhan 430070, China; ^3^Interdisciplinary Sciences Institute, Huazhong Agricultural University, Wuhan, Hubei 430070, China; ^4^State Key Laboratory of Agricultural Microbiology, College of Veterinary Medicine, Huazhong Agricultural University, Wuhan 430070, China

## Abstract

Inflammatory bowel disease (IBD), including ulcerative colitis and Crohn's disease, is mainly characterized by chronic and progressive inflammation that damages the gastrointestinal mucosa. Increasing studies have enlightened that dysregulated cell death occurs in the inflamed sites, leading to the disruption of the intestinal barrier and aggravating inflammatory response. Ferroptosis, a newly characterized form of regulated cell death, is driven by the lethal accumulation of lipid peroxides catalyzed by cellular free iron. It has been widely documented that the fundamental features of ferroptosis, including iron deposition, GSH exhaustion, GPX4 inactivation, and lipid peroxidation, are manifested in the injured gastrointestinal tract in IBD patients. Furthermore, manipulation of the critical ferroptotic genes could alter the progression, severity, or even morbidity of the experimental colitis. Herein, we critically summarize the recent advances in the field of ferroptosis, focusing on interpreting the potential engagement of ferroptosis in the pathogenesis of IBD. Moreover, we are attempting to shed light on a perspective insight into the possibility of targeting ferroptosis as novel therapeutic designs for the clinical intervention of these gastrointestinal diseases.

## 1. Introduction

Ferroptosis, a novel nonapoptotic form of regulated cell death driven by the lethal accumulation of iron-catalyzing lipid peroxides, was firstly defined by Dixon and colleagues in 2012 [1]. Since then, ferroptosis has been widely characterized in a wide spectrum of cultured cells and animal models. Distinct from other well-understood forms of regulated cell death, ferroptosis relies on the dedicated executing machinery constituted of the peroxidation of polyunsaturated fatty acid- (PUFA-) containing phospholipids (PLs), the availability of redox-active iron, and the inactivation of the lipid peroxide repair system [2]. Morphologically, ferroptosis is typically manifested by the shrinkage of mitochondria with increased membrane density and reduction in mitochondrial cristae [1, 3]. However, the hallmarks of apoptotic cell death, including plasma membrane blebbing and chromatin condensation, as well as the morphological character of necroptosis, swelling of cytoplasmic organelles, are rarely observed during ferroptotic cell death [4, 5]. Biochemically, the cytochrome c release, caspase activation, and poly(ADP-ribose) polymerase 1 cleavage (the biochemical hallmarks of apoptosis), as well as the activation of receptor-interacting protein 1, receptor-interacting protein 3, and mixed lineage kinase-like (the biochemical hallmarks of necroptosis), are dispensable for ferroptosis.

**Figure 1:**
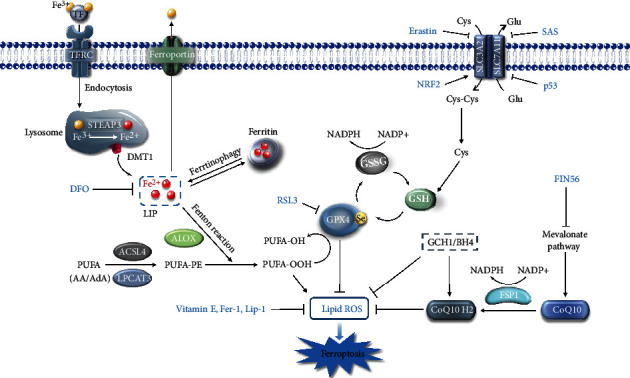
Key regulators and major signaling circuits of ferroptosis. Iron homeostasis directly influences ferroptotic sensitivity. Once absorbed by cells, ferric iron (Fe^3+^) can be reduced into ferrous iron (Fe^2+^) and chelated by ferritin or existed as labile iron. The build-up of LIP directly facilitates lipid peroxidation of PUFAs (especially AA or AdA) containing PE *via* the Fenton reaction. ACSL4 and LPCAT3 are indispensable for motivating and esterifying the PUFAs into PE for the next peroxidation. LOX families also catalyze the dioxygenation of PUFA-PE, which ultimately lead to the accumulation of lipid peroxides and cell membrane rupture. GPX4 acts as a master regulator of ferroptosis by detoxifying lipid peroxides into lipid alcohol in support of its cofactor GSH. GSH synthesis relies on multiple processes, especially the cysteine supply mediated by system X_C_^−^. Besides, the FSP1-CoQ10-NADPH axis and GCH1-BH4 axis function as lipophilic antioxidant systems parallel to the GPX4-GSH axis. The inducers and inhibitors of ferroptosis are indicated in red.

Over the years, extensive progress has been achieved in the field of ferroptosis. Specifically, the identification of potent inducers (e.g., Erastin, RSL3, FIN56, and FINO2) and specific inhibitors (e.g., DFO, Fer-1, and Lip-1) and the characterization of core regulatory components (e.g., SLC7A11, GPX4, FSP1, P53, and NRF2) support the fundamental understanding of the ferroptotic cell death (Figure 1). Moreover, emerging pieces of evidence suggest the pathological implication of dysregulated ferroptosis in the occurrence or progression of various human diseases. Herein, we summarize the recent advances of ferroptosis and dissect the potential engagement of ferroptosis in the pathogenesis of gastrointestinal diseases, attempting to elaborate the possibility of targeting ferroptosis in the therapeutic designs for the clinical intervention of gastrointestinal diseases.

## 2. Ferroptosis

Numerous pioneering studies have enlightened the fundamental characteristics of ferroptosis prior to the concept termed. In 1955, Eagle found that cystine deprivation triggers cell death with a distinct microscopic morphology compared to the deprivation of other amino acids [6, 7]. In the following decades, increasing pieces of evidence emerged that cystine deprivation leads to oxidative cell death in fibroblasts [8], embryonic cortical neurons [9, 10], and hepatocytes [11]. Besides, this cell death could be mitigated by the lipophilic antioxidant vitamin E [8] and the iron chelator deferoxamine (DFO) [12].

In 2003, Dolma and colleagues performed a lethal compound screen of genotype-selective antitumor agents and found that Erastin performs specific lethal cytotoxicity of engineered cells expressing oncogenic RAS. However, this cell death sharply differs from apoptosis. Specifically, neither caspase activation nor nuclear fragmentation was observed [4]. In 2008, two RAS-selective lethal compounds, RSL3 and RSL5, were identified in another compound screen [13]. RSL3 and RSL5 induce similar nonapoptotic cell death. Importantly, RSL3- and Erastin-mediated cell death could be alleviated by DFO and vitamin E but not by the apoptosis inhibitor z-VAD or necroptosis inhibitor necrostatin-1. Therefore, ferroptosis was coined to describe this iron- and lipid peroxidation-dependent cell death [1].

### 2.1. Ferroptosis Inducers and Inhibitors

Potent ferroptosis inducers and specific ferroptosis inhibitors have been identified during the last decade. According to the respective mechanisms of action, ferroptosis inducers are currently classified into four groups:

*(1) Glutathione (GSH) Scavengers*. Erastin depletes GSH by suppressing cystine uptake *via* restraining the cystine/glutamine antiporter system X_C_^−^ [13]. Additionally, two metabolically stable derivatives, Piperazine Erastin [14] and Imidazole Ketone Erastin [15], equip better water solubility and perform better antitumor activity in the xenograft tumor model. Likewise, sulfasalazine [16], sorafenib [17], and artesunate [18] also drive ferroptosis through exhausting GSH.

*(2) Glutathione Peroxidase 4 (GPX4) Inhibitors*. GPX4 is the sole peroxidase for catalyzing lipid peroxides into the corresponding lipid alcohols with the assistance of its cofactor GSH [19]. This class of inducers, including RSL3, Altretamine [20], and DPI17 [14], could directly inhibit GPX4.

*(3) FIN56*. FIN56 initiates ferroptosis via two distinct mechanisms. FIN56 induces GPX4 degradation in an elusive manner. Alternatively, FIN56 activates squalene synthase to deplete coenzyme Q10 (CoQ10) in the mevalonate pathway and thus impair the cellular antioxidant capacity [21].

*(4) FINO2*. FINO2 represents a unique type of organic lipophilic peroxide, which oxidizes cellular labile iron preferentially, leading to extensive oxidation of PUFAs. In addition, indirect inactivation of GPX4 also contributes to the lethal potency of FINO2 [22].

Similarly, the specific ferroptosis inhibitors could antagonize ferroptosis *via* different mechanisms. Firstly, iron chelators could confine labile free iron, leading to the deceleration of lipid peroxidation. Secondly, *β*-mercaptoethanol subverts Erastin-induced ferroptosis through forming disulfide with cystine and facilitating cystine uptake bypass system X_C_^−^ [23]. Thirdly, radical-trapping antioxidants, including vitamin E and aromatic amine-based ferrostatin-1 (Fer-1) and liproxstatin-1 (Lip-1), could halt the cascade of propagating lipid radicals and protect lipids from autoxidation [3, 24]. Fourthly, lipoxygenase (LOX) inhibitors, such as Zileuton (5-LOX inhibitor), Baicalein (12-LOX inhibitor), and NDGA (general LOX inhibitor), could counteract lipid peroxidation catalyzed by LOXs [25, 26]. Moreover, thiazolidinedione, the inhibitor of acyl-CoA synthetase long-chain family member 4 (ACSL4), represses the activation of PUFA esterification and consequently reduces the oxidizable substrates addicted by ferroptosis [27]. The identification of these inducers and inhibitors supported the primary understanding of the principal program of ferroptosis.

### 2.2. Iron and Ferroptosis

The trace element iron is critically important for tremendous biochemical processes, including oxygen transport, DNA synthesis, transcription, damage repair, redox reactions, and mitochondrial electron transport [28]. Iron also acts as a redox-active toxicant when excessive labile iron is available, which catalyzes reactive oxygen species (ROS) generation *via* the Fenton reaction. In general, most circulating iron is bound to the transferrin (TF) in the form of ferric iron (Fe^3+^). TF-Fe is captured by transferrin receptor 1 (TFR1) on the cell membrane and absorbed through endocytosis. Fe^3+^ is then escaped from the TF, reduced to ferrous iron (Fe^2+^) mediated by the endosome reductase (e.g., six-transmembrane epithelial antigen of the prostate 3), and subsequently released to the cytosol by divalent metal transporter 1 (DMT1/SLC11A2). Cytosolic iron is persistently sequestered by ferritin or transported into mitochondria for the biosynthesis of the iron-sulfur cluster or heme, two vital iron-containing cofactors for hundreds of proteins. The excessive cellular iron could be exported by ferroportin (FPN) [28].

Iron-dependent lipid peroxidation is one of the most fundamental characteristics of ferroptosis. It is thus expectable that manipulation of cellular iron metabolism or availability could change the ferroptotic sensitivity. Importantly, knockdown of iron regulatory proteins 1 and 2 (IRP1/2), the master cellular iron sensors and regulators, sharply decreases the labile iron pool (LIP) and antagonizes ferroptosis [1, 29]. Similarly, knockdown of TFR1 or ectopic overexpression of FPN impairs effective intracellular iron accumulation and abrogates ferroptosis [30, 31]. In addition, phosphorylation of heat shock protein beta-1 was reported to combat Erastin-induced ferroptosis by hindering TFR1 traffic through sustaining actin filaments and thus antagonizing iron uptake [32]. Recent studies revealed that ferritinophagy, a selective autophagy to degrade ferritin for iron motivation, plays a crucial role in ferroptosis initiation. Nuclear receptor coactivator 4 (NCOA4) acts as the selective cargo receptor responsible for recruiting and delivering ferritin to lysosomes for degradation [33]. Knockdown of autophagy-related genes (e.g., *Atg5* and *Atg7*) or ferritinophagy-specific *Ncoa4* impairs ferritinophagy and reduces the cellular labile iron, leading to ferroptotic insensitivity in various cell lines [34, 35]. Burgeoning pieces of evidence have put forward an indispensable involvement of iron in ferroptosis. At least two potential mechanisms including the Fenton reaction and activation of the enzymatic activity of iron-containing LOXs are implicated in iron facilitating the ferroptotic program [36].

### 2.3. Lipid Peroxides and Ferroptosis

As the cornerstone of cell membranes, lipid composition directly determines the biomembrane properties including fluidity, permeability, and integrity [37]. Increasing studies suggest that lipid peroxidation serves as the ultimate executor for ferroptotic cell death, although the exact mechanism is vague [38]. Lipid peroxidation leads to lipidomic alteration and compromise of the biomembrane properties (increased membrane curvature and permeability, formation of structured lipid pores, and micellization) [39, 40], which initiate exacerbating feedback to destruct biomembrane structure and dynamics. Furthermore, 4-hydroxy-2-nonenals (4-HNEs) and malondialdehydes (MDAs), two major secondary lipid peroxidation products generated by the decomposition of oxidized PUFAs, could bring out abnormal covalent modifications in proteins and nucleic acids, which could also initiate the death program [41, 42].

PUFAs, rather than saturated fatty acids or monounsaturated fatty acids (MUFAs), are preferentially oxidized by reactive radicals [26, 43, 44]. By utilizing redox lipidomic assay, it was reported that only PLs containing PUFAs (especially arachidonoyl (AA) and adrenoyl (AdA)) are the lipid precursors to undergo peroxidation preceding ferroptosis [45]. Exposure to exogenous PUFAs increases ferroptotic sensitivity. In striking contrast, supplementation of deuterated PUFAs, which are inactive to hydrogen abstraction, or administration of exogenous MUFAs (oleic acid (OA)), which competitively reduce PUFA incorporation into PLs, remodels the lipidomic composition, decelerates the accumulation of lipid peroxides, and thus potently protects cells from ferroptosis [45–47]. Furthermore, pharmacological or genetic suppression of lysophosphatidylcholine acyltransferase 3 (*Lpcat3*) and *Acsl4*, which are responsible for PUFA activation and subsequent esterification for membrane insertion, sharply prevents ferroptosis [1, 27, 45, 48, 49].

LOXs are nonheme, iron-containing dioxygenases with diverse isoforms, which oxygenate AA at different carbon positions [43, 50]. Early studies had demonstrated that deficiency or silence of arachidonate-15-lipoxygenase (*Alox15*) (encoding 12/15-LOX) or arachidonate-15-lipoxygenase type B (*Alox15b*) and arachidonate lipoxygenase 3 (*Aloxe3*) leads to dramatic resistance to GSH depletion-induced cell death [47, 51, 52]. Moreover, supplementation of 5-, 12-, and 15-hydroperoxyeicosatetraenoic acid, the production of LOX catalysis, accelerates the ferroptotic program elicited by GPX4 depletion [5]. Furthermore, inactivation of *Alox15* is not sufficient to rescue the embryonic lethality of *Gpx4*^−/−^ mice [5, 50, 53]. One potential assumption is that LOX-mediated lipid peroxidation mainly contributes to the initial build-up of the cellular lipid peroxide pool, while lipid autoxidation dominates the subsequent ferroptotic execution [25]. The alternative assumption is that other enzymes exist to catalyze lipid peroxidation bypass LOXs. Specifically, it was recently reported that NADPH-cytochrome P450 reductase (POR) and NADH-cytochrome b5 reductase (CYB5R1) could mediate the peroxidation of PUFAs of membrane PLs. By transferring electrons from the donor NADPH, POR and CYB5R1 support the generation of hydrogen peroxides, which subsequently react with iron to generate reactive hydroxyl radicals for the PUFA peroxidation [54, 55].

### 2.4. Antioxidant Defense Systems and Ferroptosis

To date, three major antioxidant defense systems have been elaborated to protect cells from ferroptosis including the GPX4-GSH axis, FSP1-CoQ10-NADPH axis, and GCH1-BH4 axis.

The selenoprotein GPX4 is the sole peroxidase that reduces the deleterious lipid peroxides to nontoxic lipid alcohols within biomembranes at the cost of oxidizing two GSH to GSSG [14, 56]. GSH, a thiol-containing tripeptide (*γ*-glutamate-cysteine-glycine) serving as an indispensable cofactor of GPX4, is recycled by NAD(P)H and glutathione reductase [57]. In this regard, disruption of GSH synthesis could initiate ferroptosis in diverse circumstances. Pharmacological inhibition of system X_C_^−^, the antiporter composed of solute carrier family 7 member 11 (SLC7A11) and solute carrier family 3 member 2 (SLC3A2), essential for exchanging intracellular glutamate and extracellular cystine, was reported to trigger ferroptosis in multiple types of cultured cells [1, 58, 59]. Notably, P53 facilitates ferroptosis by transcriptionally downregulating SLC7A11 [60]. CD8^+^ T cells enhance ferroptosis of tumor cells through releasing interferon gamma (IFN*γ*) and repressing the expression of SLC3A2 and SLC7A11 in tumor cells [61]. Moreover, nuclear factor erythroid 2-related factor 2 (NRF2) was reported to combat ferroptotic cell death *via* upregulating SLC7A11 and thus facilitating GSH synthesis [62]. Collaboratively, these studies illuminate the core role of the GPX4-GSH axis in scavenging lipid peroxides and counteracting ferroptosis.

The FSP1-CoQ10-NADPH pathway was recently characterized to compensate and synergize with the canonical GPX4-GSH pathway to detoxify lipid peroxides and defend against ferroptosis. Two independent studies based on genome-wide CRISPR-Cas9 screening for genes against ferroptosis in the absence of GPX4 coincidently identified that the flavoprotein ferroptosis suppressor protein 1 (FSP1, previously known as AIFM2) restrains ferroptosis by catalyzing the reduction of ubiquinone (namely, CoQ10) to ubiquinol in an NADPH-dependent manner [63, 64]. Intriguingly, CoQ10 is mainly generated from the mevalonate pathway, which has been demonstrated to dominate ferroptotic sensitivity. Specifically, the mevalonate-derived isopentenyl pyrophosphate can modulate the translation of selenocysteine-containing GPX4 by stabilizing the Sec-specific tRNA expression [65].

More recently, a novel mechanistic scheme accounting for cell endogenous protection from ferroptosis converges on the GCH1-BH4 axis. By utilizing whole-genome CRISPR-Cas9 screening, GTP cyclohydrolase-1 (GCH1) was nominated as a key factor to antagonize ferroptosis [66, 67]. The natural antioxidant tetrahydrobiopterin (BH4) generated by GCH1 was found to suppress ferroptosis through selectively protecting membrane PLs with two PUFA tails from oxidative degradation or alternatively promoting CoQ10 biosynthesis, which is crucial for the elimination of lipid peroxides. The proposal of the GCH1-BH4 axis provides further insights into ferroptosis resistance.

## 3. Ferroptosis and Inflammatory Bowel Disease

IBD, including ulcerative colitis (UC) [68] and Crohn's disease (CD) [69], is mainly characterized by severe gastrointestinal tract inflammation and mucosal destruction. UC is primarily disordered in the large intestine, featuring continuous mucosal inflammation beginning in the rectum and then generally extending proximally in gut tracts. Rectal bleeding, diarrhea, and abdominal pain, accompanied with ulcerations and erythema formation, are widely manifested in UC. CD principally occurs in the ileum and colon, and the typical clinical manifestations include abdominal pain, chronic diarrhea, weight loss, and fatigue. Although the exact etiology of IBD is not well understood, a combination of genetic susceptibility, harmful environmental factors, deregulated host immune system, and gut microbiota dysbiosis has been proved to be associated with the pathogenesis of IBD [70]. With the rapidly rising incidence and prevalence, IBD has emerged as a global health challenge, which will bring a considerable rise in healthcare costs [71].

The monolayer intestinal epithelial cells (IECs) covering the intestinal wall play a critical role in nutrient absorption and physical separation of the hosts from the harmful gut bacteria in the intestinal lumen. IECs are composed of multiple types of epithelial cells that differentiate from intestinal stem cells residing in the crypts, including nutrient-absorptive enterocytes, mucin glycoprotein-producing goblet cells, antimicrobial peptide-secreting Paneth cells, and hormone-secreting enteroendocrine cells [72]. It is well documented that apoptotic cell death has been observed in these different types of epithelial cells at the inflamed sites in patients with UC and CD [73, 74]. Furthermore, induction of epithelial cell apoptosis has also been evident in independent animal colitis models [75–77]. In the meantime, the expression of apoptosis-associated proteins such as the Fas cell surface death receptor, Fas ligand, BCL2 associated X, and tumor protein p53 (P53) is dramatically increased at the inflamed sites [78]. Excessive endoplasmic reticulum (ER) stress, accompanied with the overproduction of proinflammatory tumor necrosis factor alpha (TNF-*α*) released from the infiltrated macrophages, promotes epithelial cell apoptosis and further disrupts the integrity of the intestinal barrier [79–81]. Genetic ablation of critical proapoptotic components, including Puma and P53, significantly inhibits IEC apoptosis and relieves dextran sodium sulfate- (DSS-) and 2,4,6-trinitrobenzene sulfonic acid- (TNBS-) induced colitis in mice [82, 83]. Besides, other forms of regulated cell death, including necroptosis, pyroptosis, and autophagic cell death, were all evidenced to be involved in the IEC death and implicated in the colitis pathogenesis [84–87]. The IEC death would result in the collapse of the intestinal barrier, leading to the infiltration of gut bacteria and thus aggravating the inflammation.

Ferroptosis is a newly characterized form of regulated cell death. As mentioned above, iron overload, GSH depletion, GPX4 inactivation, and lipid peroxidation constitute the fundamental features of ferroptosis. Direct and indirect studies have enlightened a tight link between ferroptosis and intestinal diseases.

### 3.1. Iron and IBD

The earlier study suggested that the clinical symptoms of IBD include iron deficiency and anemia due to bleeding and malabsorption, which seriously influence individual health [88]. Oral iron administration has been clinically used to improve the IBD patients with iron deficiency anemia [89]. However, excessive iron administration leads to iron overload in the intestinal tract, resulting in the dysregulated production of ROS and disturbing the gut microbiota, which may exacerbate the illness of IBD [90–92]. Hereditary hemochromatosis is an iron overload disease due to recessive mutations in the hemochromatosis gene (*Hfe*), and it is characterized by increased iron absorption in the proximal intestine [93]. It was reported that some patients with hereditary hemochromatosis exhibit histologic abnormalities accompanied with increased intraepithelial neutrophil infiltration and lamina propria lymphocyte infiltration in the intestinal tract [94]. MDA was elevated in the colon tissue of the *Hfe* knockout mice, the murine model of human hereditary hemochromatosis, suggesting iron overload facilitates oxidative damage in the gut [95]. Importantly, *Hfe* knockout mice are more susceptible to the development of experimental colitis, as evidenced by more severe rectal bleeding and diarrhea, higher colonic mucosal injury with frequent ulcerations, and a markedly increased loss of villus integrity [96]. Collaboratively, these studies thus highlight a pathological role of iron overload in the development of colitis. It is supposed that iron deposition in the intestine results in severe oxidative stress and facilitates lipid peroxidation through the Fenton reaction, which is probably pathogenic for colitis [97–99]. However, it is still elusive whether iron deposition initiates ferroptosis and is responsible for aggravated IEC death, mucosal damage, and intestinal inflammation. Ablin and colleagues reported that oral administration of iron chelator deferiprone (DFP) protects against experimental colitis and gastric ulceration in rats [100]. However, another study raised an opposite argument that oral iron supplementation in young rats has a beneficial effect on the prevention of TNBS-induced colitis [101].

### 3.2. GSH and IBD

GSH depletion is a critical signature of ferroptosis. It is now well understood that GSH exhaustion and GPX4 inactivation are widely observed in the inflamed mucosa from patients with IBD and in experimental animal models of colitis [102, 103]. The elevated oxidative insult in inflamed sites exhausts the endogenous GSH, while the reduced plasma cysteine and decreased enzymatic activity of mucosal *γ*-glutamylcysteine synthetase or *γ*-glutamyl transferase essential for GSH biosynthesis decelerate the de novo synthesis of GSH in patients with CD and UC [104]. Administration of the specific inhibitor of *γ*-glutamylcysteine synthetase, the rate-limiting enzyme for GSH synthesis, leads to a rapid decline of GSH and a substantial loss of the epithelial cells in the jejunal and colonic mucosa [105]. On the contrary, replenishment of GSH through administration of GSH [106], GSH ester [106], N-acetylcysteine [107], or L-cysteine [108] could restore the intestinal GSH abundance and significantly improve colonic health. GSH could confer the cellular antioxidative capacity by directly scavenging ROS and supporting the enzymatic activity of glutathione S-transferases to defend against oxidative stress, which are protective for the gastrointestinal tract from chronic inflammation [109].

### 3.3. GPX4 and IBD

Antioxidant enzyme GPX4 is responsible for scavenging lipid hydroperoxides and antagonizing ferroptosis [5]. Early studies indicated a genetic association between GPX4 and CD by using a meta-analysis of GWAS [110, 111]. Reduced GPX4 activity accompanied with elevated lipid peroxidation was characterized in the intestinal epithelium in patients with CD. A diet enriched in PUFAs, but not saturated fatty acids, induces focal enteritis in IEC-specific *Gpx4*^+/−^ mice. More strikingly, IEC *Gpx4*^+/−^ mice are more susceptible to colonic inflammation induced by DSS, as compared to the wild-type littermates, highlighting the notion that GPX4 is crucial for maintaining gut homeostasis by protecting from lipid peroxidation [112]. Furthermore, an increasing number of studies have suggested a tight association between IBD and the secondary metabolites of lipid peroxidation such as MDA and 4-HNE [112, 113]. The content of AA, one of the most oxidizable PUFAs preferentially for lipid peroxidation, is markedly elevated in PLs of the colonic mucosa in patients with UC [114, 115]. Therefore, the inactivation of GPX4 and the elevation of lipid peroxides indicate the possibility that GPX4 determines gut homeostasis by antagonizing lipid peroxidation. Moreover, a reduced level of serum selenium was evidenced to be associated with the pathogenesis of UC and CD [116, 117]. Selenium deficiency in mice exacerbates intestinal injury [118], while selenium supplementation has been reported to be protective in IBD patients [119–122]. It is still elusive whether selenium supplementation ameliorating intestinal injury depends on the transcriptional activation of GPX4 or not [65, 123]. In addition, selenium supplementation in cultured Caco-2 cells could significantly prevent the transport of lipid hydroperoxides and thus decline cellular lipid peroxidation [124].

### 3.4. LOXs and IBD

LOXs catalyze the production of lipid hydroperoxides and drive ferroptotic cell death [45, 50]. Several LOX isoforms have been identified to be involved in the pathogenesis of IBD. More specifically, the levels of *Alox5* and *Alox15* are upregulated in the colonic mucosa in patients with IBD and in the experimental colitis mouse model, respectively [125, 126]. Systemic deletion of *Alox15* suppresses the production of lipid peroxidation metabolite 12-hydroxyeicosatetraenoic acid, stabilizes the tight junction protein ZO-1 and maintains the intestinal barrier integrity, decreases macrophage infiltration, and reduces the expression of proinflammatory genes, thus alleviating colonic damage in DSS-induced experimental colitis in mice. Conversely, transgenic overexpression of human *Alox15* renders mice more susceptible to DSS-induced colitis [127]. Similarly, deficiency of *Alox15* was reported to protect mice from DNBS-induced mucosal injury. Phosphatidylethanolamine-binding protein 1 (PEBP1) is a master regulatory molecule for 15-LOX by dominating the substrate specificity of 15-LOX to PUFA-phosphatidylethanolamines (PUFA-PE), facilitating the generation of lipid peroxides [128]. It suggested a positive correlation between the PEBP1 expression and the severity of IBD. More importantly, PEBP1 deficiency protects mice from DSS- or TNBS-induced colitis and accelerates mucosal recovery from injury [129]. Similarly, the supplementation of Zileuton, the potent 5-LOX inhibitor, maintains the tight junction proteins to prevent the decrease in the tight junctional permselectivity induced by TNBS [130]. Other 5-LOX-selective inhibitors, including A-64077 and MK-0591, could alleviate the inflammatory status in the colon of UC patients [131–134]. Collectively, these studies suggest a critical role of LOXs and their metabolites in determining gut inflammation and intestinal homeostasis.

### 3.5. GCH1/BH4 and IBD

Folate, also known as folic acid and vitamin B_9_, is regarded as a major endogenous antioxidant to defend against oxidative insults [135]. It is well recognized that folate is commonly deficient in patients with UC due to malabsorption [136, 137]. Administration of folate or its metabolic precursor BH4 was evidenced to relieve colitis-related tissue damage, detrimental inflammation, and malignant tumorigenesis [138, 139]. GCH1-mediated BH4 biosynthesis is crucial for ferroptosis resistance by remodeling lipidomic composition and suppressing lipid peroxidation [67, 140]. Ionizing radiation decreases BH4 levels and increases superoxide anion accumulation in patients and rats after radiotherapy due to the downregulation of GCH1. BH4 supplementation could prevent intestinal ischemia, improve vascular endothelial function, relieve intestinal villus injury, and thus alleviate radiation enteritis [141]. Collectively, these studies thus indicate an essential role of GCH1-mediated BH4 and folate biosynthesis in maintaining intestinal homeostasis.

### 3.6. The Emergence of Ferroptosis in Intestinal Diseases

As summarized above, the fundamental features of ferroptosis, including iron deposition, accumulation of lipid peroxidation, GSH exhaustion, GPX4 inactivation, and LOX upregulation, have been elucidated to be implicated in the pathogenesis of IBD. Additionally, recent studies have enlightened a direct engagement of ferroptosis in the pathogenesis of IBD. The ER stress signaling is involved in the IEC ferroptosis during chemical colitis, as evidenced by the elevated expression of ER stress-associated G protein-coupled receptor 78, phosphorylated eukaryotic initiation factor 2, activating transcription factor 4, and C/EBP homologous protein. Specifically, selective inhibition of protein kinase RNA-like endoplasmic reticulum kinase, the critical stress sensor of ER stress signaling, sharply reduces IEC ferroptosis and significantly ameliorates experimental colitis. NF-*κ*B activation could protect against IEC cell death during acute intestinal inflammation. Importantly, specific deletion of the nuclear factor kappa B p65 subunit (NF-*κ*Bp65) in IECs leads to upregulated ER stress-mediated ferroptosis and aggravates DSS-induced colitis in mice [142]. More importantly, Fer-1, the specific inhibitor for ferroptosis, could ameliorate DSS-induced colitis [142]. Other well-characterized ferroptosis inhibitors, including Lip-1, iron chelator DFP, and antioxidant butylated hydroxyanisole, could all decelerate ferroptotic hallmarks and alleviate colonic damage [143]. Similarly, curculigoside, a natural ingredient from *Curculigo orchioides* Gaertn with multiple biological activities, was recently identified to attenuate DSS-induced UC in mice. Mechanistically, curculigoside supports GPX4 expression and thus protects against ferroptotic cell death in a selenium-dependent manner [144]. These research studies collaboratively put forward the notion of the pathological engagement of ferroptosis in colitis.

ACSL4 is responsible for the esterification of AA and AdA into PLs to facilitate the subsequent peroxidation. Genetic and pharmacological inhibition of ACSL4 protects cells from lipid peroxidation and ferroptosis [27, 45, 49]. It was previously reported that ACSL4 is upregulated in the ileum and colon of patients with CD and UC [145] and in DSS-induced experimental colitis in mice [143]. Intestinal ischemia/reperfusion injury is a life-threatening condition associated with a high mortality rate, which commonly occurs in numerous clinical pathologies such as small intestinal volvulus, acute mesenteric ischemia, shock, trauma, and small bowel transplantation [146]. Recently, Li and colleagues reported that ACSL4 is sharply induced in ischemic intestines compared with normal intestines, possibly *via* the transcription factor special protein 1. More importantly, the core hallmarks of ferroptosis, including iron deposition, reduction of the GPX4 activity and GSH level, rupture of the outer mitochondrial membrane, and accumulation of lipid peroxidation, are manifested in the intestine after reperfusion. The typical ferroptosis inhibitor Lip-1 could strongly block lipid peroxidation and suppress cell death both *in vitro* and *in vivo*. Similarly, oral administration of rosiglitazone could inhibit ACSL4, suppress lipid peroxidation, and thus alleviate ischemia/reperfusion-related mucosal injury. Moreover, siRNA-mediated ACSL4 silence also protects Caco-2 cells from hypoxia/reoxygenation-induced lipid peroxidation and cell death [147]. Therefore, this study thus shed new light on the pathological engagement of ACSL4-mediated ferroptosis in intestinal ischemia/reperfusion injury.

### 3.7. Other Ferroptosis Regulators in Intestinal Diseases

Iron overload, lipid peroxidation, GSH depletion, and GPX4 inactivation constitute the fundamental features of ferroptosis. Besides, there are other ferroptosis regulators that have been evidenced to be associated with the pathogenesis or progression of intestinal diseases.

P53, one of the most famous tumor suppressors, is mutated in many types of human cancers. Specifically, P53 is mutated in about 55%-60% of human colorectal cancers, and its mutations are associated with a poor prognosis in colorectal cancers [148]. Besides colorectal cancer, a high frequency of P53 mutations was also reported in patients with chronic UC [149]. In response to diverse stimuli, P53 is stabilized to mediate metabolic reprogramming, cell cycle arrest, cellular senescence, and even cell death [150]. Genetic depletion of *P53* leads to a significantly reduced cell death of IECs, but the colonic inflammation is not altered in a murine colitis model [83]. Other studies indicated that the knockout of *P53* leads to comparable histopathologic changes of chronic colitis. However, a significantly greater incidence and multiplicity of cancers are observed during P53 deficiency [151–153]. Recently, it was reported that P53 suppresses cystine uptake, disturbs GSH biosynthesis, and thus sensitizes cells to ferroptosis. Mechanistically, P53 transcriptionally restrains the expression of cystine/glutamate antiporter subunit SLC7A11 [60, 154, 155]. Alternatively, P53 could facilitate the ferroptotic program by directly activating its target gene spermidine/spermine N1-acetyltransferase 1 and the downstream *Alox15* [156] or through transcriptionally upregulating the mitochondrial glutaminase 2 [30]. However, other studies suggest an opposite notion that P53 may inhibit ferroptotic cell death through dipeptidyl peptidase-4 [157] or cyclin-dependent kinase inhibitor 1A [158]. Whether P53-modulated ferroptotic sensitivity accounts for the pathogenesis or malignancy of colitis or not is still ambiguous.

The transcription factor NRF2, encoded by the *Nfe2l2* gene, plays a central role in the cytoprotective antioxidant system in response to a variety of oxidative, inflammatory, and metabolic stresses. NRF2 dominates the basal and induced expression of a series of antioxidant response element-dependent genes [159]. The increased severity of DSS-induced colitis and the elevated susceptibility of colitis-associated colorectal cancer in NRF2-ablated mice were found to be associated with the decreased expression of antioxidant genes and detoxifying enzymes, as well as the increased expression of proinflammatory cytokines [160, 161]. Among them, heme oxygenase-1 (HO-1) presents pronounced anti-inflammatory and antioxidative properties in protecting mice from colitis-associated inflammatory injury and oxidative stress [162, 163]. As the main antioxidant axis, NRF2/HO-1 also dominates ferroptotic sensitivity. Ectopic expression or activation of NRF2 counteracts ferroptosis, whereas knockdown of NRF2 elevates the ferroptotic sensitivity in response to diverse ferroptosis inducers [164–166]. It is thus expectable that a variety of compounds that activate NRF2 could alleviate colitis-associated mucosal damage and colonic inflammation [167].

In addition, other ferroptosis regulators, including NADPH oxidases [168–171] and CD44 [172–175], are evidenced to be associated with the pathogenesis of IBD in patients or in colitis models. These proteins, including P53, NRF2, NADPH oxidases, and CD44, are all multifaceted. Thus, the exact involvement of these molecules in mediating ferroptotic regulation in colitis needs further investigation.

## 4. Conclusive Remarks and Perspective

IBD is increasing worldwide and has become a global disease in both developed regions and developing countries. The increasing medicinal cost and substantial elevation in the risk of colorectal cancer are greatly affecting the life quality of patients and families. Although the exact pathogenesis of IBD is poorly defined, multiple lines of evidence indicate that genetic susceptibility, deleterious environmental factors, and an imbalanced gut microbial ecosystem could impinge on the gut homeostasis and thus facilitate inflammatory response [195]. Uncontrolled cell death has been widely observed in the diseased mucosa in patients and animal models, which could disturb the tight junction of the intestinal barrier and then aggravate the inflammation by releasing the gut microorganisms.

Ferroptosis is a newly identified form of regulated cell death. Iron overload, GSH exhaustion, GPX4 inactivation, and lipid peroxidation are the major features of ferroptosis. Dysregulated ferroptosis has been evidenced to be implicated in the pathogenesis and progression of many human diseases [196]. Furthermore, targeting induction of ferroptosis provides a potential therapeutic strategy for the clinical intervention of cancers, especially the other traditional therapy-resistant cancers [197, 198]. As mentioned above, the major features of ferroptosis have been extensively observed in the diseased mucosa in patients and animal models. Importantly, genetic or pharmacological manipulation of ferroptosis-related genes could alter the incidence, severity, or progression of the experimental colitis by using the corresponding murine models. More directly, some potent ferroptosis inhibitors, including iron chelators, GSH or GSH derivate GSH ester, selenium, LOX inhibitors, folate, or BH4, could decline lipid peroxidation and alleviate colitis-associated intestinal injury (Table 1). Moreover, Fer-1 and Lip-1, two specific inhibitors of ferroptosis, could relieve colitis in murine models. On the contrary, the ferroptosis sensitizers, including iron, *γ*-glutamylcysteine synthetase inhibitor BSO, and dietary PUFAs, could accelerate lipid peroxidation and aggravate colitis. Collaboratively, these studies thus highlight the critical importance of dysregulated ferroptosis in the pathogenesis of IBD.

**Figure 2:**
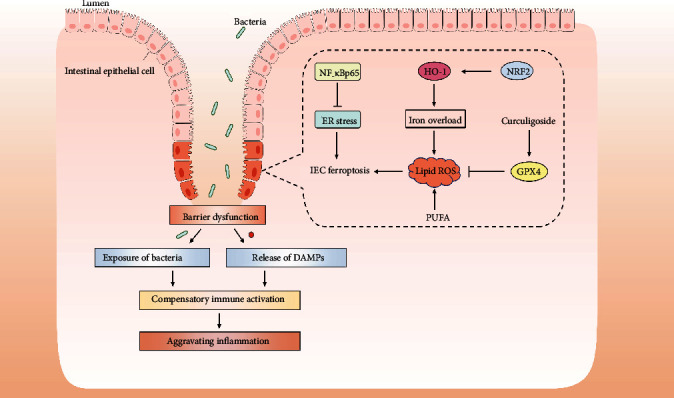
Emerging role of ferroptosis in inflammatory bowel disease. Ferroptosis has been directly implicated in the pathogenesis of IBD in recent studies. IEC ferroptosis seems to promote disruptions in epithelial barrier function, thereby allowing luminal antigens and cellular damage-associated molecular patterns (DAMPs) released into the bowel wall. Subsequently, immune cells and cytokine production are activated excessively, which in turn lead to intestinal inflammation and epithelial injury.

It should not be ignored that abnormalities of both the innate and adaptive immune responses against harmful intestinal microorganisms, antigens, or extrinsic pathogens play important roles in the pathogenesis of IBD. The healthy mucosa contains a delicate balance of innate lymphoid cells, macrophages, neutrophils, and dendritic cells, as well as the adaptive immune response associated with T and B cells. The hyperactivation of the intestinal immune system due to the epithelial cell death and intestinal barrier disruption leads to the subsequently excessive secretion of proinflammatory cytokines and chemokines, which could result in secondary damage to the intestinal mucosa and a vicious cycle [199, 200]. Furthermore, previous studies indicated resistance to cell death of lamina propria lymphocytes in inflamed tissues in UC patients due to the altered expression of cell death-associated proteins [201–203]. Therefore, it is supposed that the IEC ferroptosis leads to intestinal barrier disruption, gut microorganism release, and hyperactivation of intestinal immune response, resulting in aggravation of colitis-associated mucosal injury. Furthermore, the ferroptotic IECs would release some immunogenic molecules, which may further facilitate local inflammation (Figure 2) [204]. However, besides IECs, whether intestinal immune cells undergo ferroptosis in the pathogenesis of intestinal injury or not is elusive. If so, whether this ferroptosis in certain types of intestinal immune cells contributes to the pathogenesis or progression of intestinal diseases or not needs more investigations.

Regarding the beneficial effect of diverse ferroptosis inhibitors in relieving colitis-associated tissue injury (Table 1), it is of great therapeutic potential for selective manipulation of ferroptosis in the prevention and intervention of colitis. Therefore, more extensive investigations are needed to further dissect the exact implication of ferroptosis in the pathogenesis of IBD and other related intestinal diseases. Specifically, to dissect the detailed underlying molecular mechanism for which ferroptosis mediates mucosal damage in inflamed tissues, to explore the specific types of epithelial cells in which dysregulated ferroptosis occurs leading to the hyperactivation of intestinal inflammation, and to identify the more selective and potent ferroptosis inhibitors with lower side effects for pharmacological intervention of IBD will help to obtain the full aerial view of ferroptosis and provide some future translational applications.

## Figures and Tables

**Table 1:** Promising molecules targeting ferroptosis in IBD.

Effect	Drug	Target	Mechanisms	Model	References
Inhibitors	Curculigoside	GPX4	Increases selenium sensitivity and promotes GPX4 expression	IEC-6 cells, UC mice	[144]
	NAC	GSH	Increases mucosal GSH levels	UC rats	[107]
	SAM	GSH	Serves as a precursor for GSH biosynthesis and antagonizes ROS	UC mice	[176]
	PTCA	GSH	Functions as a cysteine prodrug that stimulates GSH biosynthesis	UC mice	[176]
	DFP	Iron	Chelates excessive free iron and suppresses iron-dependent lipid peroxidation	UC mice	[100, 143, 177]
	DFO	Iron	Chelates excessive free iron and suppresses iron-dependent lipid peroxidation	UC mice	[142, 143, 177]
	Maltol	Iron	Oxyradical scavenger and/or iron chelation	UC rats	[177]
	Fer-1	ROS	Blocks lipid peroxidation and restrains ROS overgeneration	UC mice	[142, 143]
	Lip-1	ROS	Lipophilic antioxidants	UC mice	[143]
	Simvastatin	ROS	Decreases the TNF-*α* level and reduces oxidative stress	IECs, UC mice, UC rats	[178–180]
	Rosuvastatin	ROS	Decreases the TNF-*α* level and reduces oxidative stress	UC mice, UC rats	[180, 181]
	Vitamin E	ROS	Protects against lipid peroxidation and scavenges free radicals	UC rats	[98, 99, 182, 183]
	TMG	ROS	Protects against lipid peroxidation and scavenges free radicals	UC rats	[184, 185]
	AA	ROS	Increases the activities of GPX and reduces oxidative stress	UC mice	[186, 187]
	5-ASA	ROS	Scavenges oxygen-derived free radicals	IBD patients	[188, 189]
	CoQ10	ROS	Antioxidant and anti-inflammatory properties	UC rats	[190, 191]
	Melatonin	ROS	Antioxidant and anti-inflammatory properties	UC rats, UC mice	[192, 193]
	LS	ROS	Reduces lipid peroxidation and restores the levels of innate antioxidants	UC mice	[113]
	BH4	ROS	Reduces oxidative stress and rebalances lipid signaling *via* alkylglycerol monooxygenase	UC mice	[139]
	Zileuton	5-LOX	Functions as a 5-LOX inhibitor to increase PGE2 levels and reduces myeloperoxidase activity	IBD patients, UC rats	[134, 194]

Inducers	Oral iron	Iron	Exacerbates oxidative stress through the Fenton reaction	UC rats	[98, 182]

Abbreviations: AA: acetic acid; AA: ascorbic acid; BH4: tetrahydrobiopterin; CoQ10: coenzyme Q10; DFP: deferiprone; DFO: deferoxamine; DSS: dextran sodium sulfate; Fer-1: ferrostatin-1; Lip-1: liproxstatin-1; LS: *Lagerstroemia speciosa* leaves; NAC: N-acetylcysteine; PTCA: 2(R,S)-n-propylthiazolidine-4(R)-carboxylic acid; SAM: S-adenosylmethionine; TMG: vitamin E derivative, 2-(alpha-D-glucopyranosyl)methyl-2,5,7,8-tetra-methylchroman-6-ol; TNBS: trinitrobenzene sulfonic acid; 5-ASA: 5-aminosalicylic acid.

## Abbreviations

4-HNEs: 4-Hydroxy-2-nonenals
5-ASA: 5-Aminosalicylic acid
AA: Arachidonoyl
ACSL4: Acyl-CoA synthetase long-chain family member 4
AdA: Adrenoyl
ALOX: Arachidonate lipoxygenase
BH4: Tetrahydrobiopterin
CD: Crohn's disease
CoQ10: Coenzyme Q10
CYB5R1: NADH-cytochrome b5 reductase
DAMPs: Damage-associated molecular patterns
DFO: Deferoxamine
DFP: Deferiprone
DMT1: Divalent metal transporter 1
DSS: Dextran sodium sulfate
ER: Endoplasmic reticulum
Fe^2+^: Ferrous iron
Fe^3+^: Ferric iron
Fer-1: Ferrostatin-1
FPN: Ferroportin
FSP1: Ferroptosis suppressor protein 1
GCH1: GTP cyclohydrolase-1
GPX4: Glutathione peroxidase 4
GSH: Glutathione
HFE: Hemochromatosis gene
HO-1: Heme oxygenase-1
IBD: Inflammatory bowel disease
IECs: Intestinal epithelial cells
IFN*γ*: Interferon gamma
IRP1/2: Iron regulatory proteins 1 and 2
LIP: Labile iron pool
Lip-1: Liproxstatin-1
LOXs: Lipoxygenases
LPCAT3: Lysophosphatidylcholine acyltransferase 3
MDAs: Malondialdehydes
MUFAs: Monounsaturated fatty acids
NCOA4: Nuclear receptor coactivator 4
NF-*κ*B: Nuclear factor kappa B
NRF2: Nuclear factor erythroid 2-related factor 2
P53: Tumor protein p53
PE: Phosphatidylethanolamines
PEBP1: PE-binding protein 1
PLs: Phospholipids
POR: P450 reductase
PUFAs: Polyunsaturated fatty acids
ROS: Reactive oxygen species
SLC3A2: Solute carrier family 3 member 2
SLC7A11: Solute carrier family 7 member 11
TF: Transferrin
TFR1: Transferrin receptor 1
TNBS: 2,4,6-Trinitrobenzene sulfonic acid
TNF-*α*: Tumor necrosis factor alpha
UC: Ulcerative colitis.
